# An Assessment of Food Safety Needs of Restaurants in Owerri, Imo State, Nigeria

**DOI:** 10.3390/ijerph10083296

**Published:** 2013-08-02

**Authors:** Sylvester N. Onyeneho, Craig W. Hedberg

**Affiliations:** Division of Environmental Health Sciences, University of Minnesota, 420 Delaware Street SE, MMC807, Minneapolis, MN 55455, USA; E-Mails: onye0005@umn.edu (S.N.O.); hedbe005@umn.edu (C.W.H.); Tel.: +1-952-484-6796 (S.N.O.); +1-612-626-4757 (C.W.H.); Fax: +1-952-707-9684 (S.N.O.); +1-612-626-6931 (C.W.H.)

**Keywords:** restaurants, food safety, foodborne pathogens, hygiene, cross-contamination

## Abstract

One hundred and forty five head chefs and catering managers of restaurants in Owerri, Nigeria were surveyed to establish their knowledge of food safety hazards and control measures. Face-to-face interviews were conducted and data collected on their knowledge of risk perception, food handling practices, temperature control, foodborne pathogens, and personal hygiene. Ninety-two percent reported that they clean and sanitize food equipment and contact surfaces while 37% engaged in cross-contamination practices. Forty-nine percent reported that they would allow a sick person to handle food. Only 70% reported that they always washed their hands while 6% said that they continued cooking after cracking raw eggs. All respondents said that they washed their hands after handling raw meat, chicken or fish. About 35% lacked knowledge of ideal refrigeration temperature while 6% could not adjust refrigerator temperature. Only 40%, 28%, and 21% had knowledge of *Salmonella*, *E. coli*, and Hepatitis A, respectively while 8% and 3% had knowledge of *Listeria* and *Vibrio* respectively, as pathogens. Open markets and private bore holes supplied most of their foods and water, respectively. Pearson’s Correlation Coefficient analysis revealed almost perfect linear relationship between education and knowledge of pathogens (*r* = 0.999), cooking school attendance and food safety knowledge (*r* = 0.992), and class of restaurant and food safety knowledge (*r* = 0.878). The lack of current knowledge of food safety among restaurant staff highlights increased risk associated with fast foods and restaurants in Owerri.

## 1. Introduction

Although the safety of foods served in restaurants in Nigeria has been an ongoing concern, the unhygienic environments where some restaurants are located contribute to transmission of foodborne diseases. Health experts in Nigeria have said that the shortage of water may affect the success of any safety campaigns. According to the WHO/UNICEF Joint Monitoring Program (JMP) for water and sanitation [1], only 58 percent of the country’s 160 million people have access to potable water. Food safety issues in Africa are mostly centered on illnesses that are linked to poor hygiene but food hygiene in homes, schools and markets remains an area of concern. Although outbreaks are frequent in the African region, individual countries have done little to implement surveillance systems for food-borne diseases [2]. Some of the challenges to any surveillance system in the country include the fact that in many parts of Nigeria, food poisoning is usually associated with evil spirit, malice or curses [3]. Also, the culture of self-prescription prevents cases from being treated at hospitals. Symptoms of foodborne illnesses such as vomiting, diarrhea, fever are regarded as “common diseases” and everyone seems to know the cure and requires only going to the “chemist” to purchase self-prescribed medication. There is lack of proper monitoring and supervision by food safety officers and the enforcement of food hygiene regulations. In recognition of the importance of food safety as an essential factor for achieving a high level of health for all Nigerians, the Government of Nigeria launched the National Policy of Food Hygiene and Safety in 2000 as an integral part of the Nigerian National Health Policy. The overall goal of this policy was the attainment of a high level of food hygiene and safety practices that will promote health, control food-borne diseases, minimize and finally eliminate the risk of diseases related to poor food hygiene and safety [2]. In most developing countries including Nigeria, urban adults eat food from street vendors regularly because it is easily available, affordable and usually fresh. Street-vended foods may pose significant public health problems due to lack of basic infrastructure and services, such as potable water supplies, their temporary nature, and poor knowledge of basic food safety measures [4]. Many of the food vendors are itinerant in nature and vary from mobile carts to fixed stalls and food centers. The operators push their food-laden carts, wheel-barrows or specially-designed bicycles from one location to another often “parking” under tree shades to serve their customers. Food vending and food hawking have been an integral and important part of the business culture of Igbo people who inhabit Imo State. They provide a source of inexpensive, convenient and often nutritious food for urban and rural poor. They are also a source of attractive and varied food for tourists and the economically advantaged. Most importantly, they are a major source of income for a vast number of persons, a chance for self-employment, and the opportunity to develop business skills with low capital investment. Besides offering business opportunities for developing entrepreneurs, the sale of street foods can make a sizeable contribution to the economies of developing countries [5].

One obstacle to food safety in Nigeria is refuse disposal and lack of toilet facilities for the customers. Most of the eating stalls around markets in Nigeria are characterized by unsanitary conditions, including poor water supply and poor drainage systems, unsanitary waste disposal and overcrowding, resulting in poor personal and environmental hygiene [6]. Another area of food safety concern is the source foods, and ingredients supply. Most of these establishments purchase their raw materials and ingredients in the open markets. Foods, especially meat, fish and ingredients are often displayed openly on tables and on the ground in very poor sanitary environments, while unwashed fingers are used to feel foodstuffs and ingredients for texture and to ascertain the adequacy of manual grinding. The prevalence of flies at the markets and the apparent lack of facilities for food protection suggest a high potential for contamination in preparation, facilities and infrastructure [7].

Although Nigeria has no official foodborne disease surveillance system, cases of food poisoning resulting in deaths and hospitalizations have been reported. An outbreak of food poisoning in Ibadan, Nigeria, claimed about 20 lives and a new phage type U282 of *Salmonella typhimurium*, the causative organism, was isolated from a sandwich filling [8]. Cases of food poisoning among three families in Kano due to yam flour consumption were reported and investigations indicated that the use of certain lethal preservatives for the processing of the yam flour might be responsible [9]. Another food poisoning report attributed to yam flour consumption in five families in Ilorin, central Nigeria was also reported [10]. The rising cases of food poisoning in Nigeria were linked to the misuse and abuse of agrochemicals and pesticides on grains and other agricultural products. The report stated that these chemicals were wrongly applied and abused in preventing pests. Over three million cases of acute food poisoning and twenty-thousand deaths occur annually due to exposure of food pesticides. An acute onset of gastrointestinal symptoms among people who had attended and eaten at a burial ceremony resulted in 60 case patients and 3 deaths [11]. The WHO estimates that more than 200,000 people die of food poisoning annually in Nigeria from foodborne pathogens (especially *E. coli* and *Salmonella*). The deaths were caused by contaminated foods through improper processing, preservation and service [12].

Owerri is the capital city of Imo State in southeastern Nigeria. Owerri had many registered hotels and school cafeterias but, hundreds of eating houses, food canteens, food kiosks and other food vending stalls were scattered in and around the city. Most of the hotels operated restaurants while most of the schools had cafeterias. However, street kiosks with poor hygiene and food handling practices are often the sources of lunch for school children in Nigeria who attend schools that do not operate school-based cafeterias. Despite these benefits, food safety is still a major concern with street foods. Most of these foods are generally prepared and sold under unhygienic conditions, with limited access to safe water, sanitary services, or garbage disposal facilities. Owerri lies within an area with a modified rainforest climate and this temperature-humidity combination favors year-round luxuriant microbial growth. Food pathogens of concern to public health include pathogenic strains of bacteria, viruses, helminthes, protozoa and algae, and certain toxic products they may produce. Foodborne infections are caused when microorganisms are ingested, and these can multiply in the human body. Intoxications result when microbial or naturally occurring toxins are consumed in contaminated foods [13]. Because restaurants with food safety-certified kitchen managers are less likely to be associated with foodborne illness outbreaks than restaurants without these managers and, because restaurants with food safety-certified kitchen managers are less likely to have critical violations on their food safety inspections than restaurants without these managers [14], this investigation was undertaken with the following objectives in mind:

(i) To ascertain the areas where food safety knowledge is lacking among restaurant managers and head cooks, and (ii) To identify the main areas in these eating places where hygiene and food safety need improvement.

## 2. Materials and Methods

### 2.1. Subjects

Managers and head chefs responsible for food hygiene of four classes of restaurants in the city of Owerri, the capital of Imo state, Nigeria were surveyed to establish their knowledge of food safety protocols. Face-to-face interviews were conducted to collect data on their knowledge of five elements of food safety: risk perception, food handling practices, temperature control, foodborne disease pathogens, and personal hygiene. The sources of their food and water supplies were also determined. Managers of four classes of restaurants were surveyed for this study: Class A—restaurants in major hotels; Class B—school cafeterias; Class C—regular/fast food type; and Class D—“bukas” or “bukaterias” (food kiosks, roadside food sellers and itinerant food sellers or food hawkers). Clearance to use human subjects for this study (head chefs locally known as “head cooks and/or hotel managers” as well as cafeteria managers of some schools in Owerri was obtained from the Institutional Review Board (IRB) of the University of Minnesota. Restaurants in government-registered hotels, school cafeterias, fast food-type eating houses, food canteens, food kiosks and other food vending stalls in Owerri were included in the study.

### 2.2. Survey Administration

For this survey, the city of Owerri was divided into six zones: central, north, south, east, west, and elementary/secondary schools. Face-to-face interviews of managers of restaurants and school cafeterias in each zone were conducted to obtain data on training, food handling, personal hygiene, temperature control, food preparation, the concept and application of HACCP, and knowledge of relevant bacterial pathogens. Most of the thirty-three survey questions were single-select, multiple choice format. The questions were selected from the 2006 U.S. FDA/FSIS Food Safety Survey Topline Frequency Report [15]. Some of the questions were modified to reflect the local terminologies, foods, and ingredients normally used for cooking in Nigeria. The questionnaire was divided into the following six sections: A: Risk Perception; B: Food Handling Practices; C: Temperature Control; D: Microorganisms, Pests and Supplies; E: Personnel Hygiene and Facilities and F: Demographics. Data was collected only from consenting restaurants that did not require any pre-conditions (Table 1).

**Table 1:** Demographic Data.

Restaurant Class	Surveys Completed	Males (%)	Females (%)	College Ed. (%)	Cooking Sch. Ed. (%)	Average Restaurant Experience	Average Employees	Average Weekly Customers
A (Hotel-based)	40	75	25	100	100	10	20	1,500
B (School Cafe)	30	0	100	100	100	15	25	2,500
C (Fast-food )	50	29	71	30	0	25	10	4,000
D (Bukaterias)	25	0	100	0	0	8	3	600
Total	145							

## 3. Results

Two hundred survey questionnaires and consent forms were distributed to the managers but one hundred and forty-five consented questionnaires (73%) were returned (Table 1). None of the respondents provided his or her age but all marked “adult” as a response. In class A (restaurants in major hotels), 75% of the managers were males and 25% were females. All were educated (to a minimum technical college level), all went to cooking school, and had completed a Food Hygiene course. The average restaurant experience of managers was 10 years. The restaurants had an average of 20 employees and served an average of 1,500 customers per week. In class B restaurants (school cafeterias), all the managers were female, educated and had cooking school and Food Hygiene training credentials. Managers averaged 15 years restaurant experience. Schools had an average of 25 employees and served an average of 2,500 school children every week. The owners/mangers of class C (fast-food type restaurants) were 10 males and 25 females. Fifty-seven percent had high school education and 30% indicated they had technical college education. No manager in this category attended Cooking School or Food Hygiene training. Their average restaurant experience was 25 years. These restaurants had an average of 10 employees and served an average of 4,000 customers per week. All the managers/owners of class D restaurants or “bukaterias”, were females, and had only primary school education. None of them attended Cooking School or Food Hygiene training. They averaged 8 years of restaurant experience. They had an average of three employees and served an average of 600 customers per week.

### 3.1. Risk Perception

Over 72% of the respondents reported that it was very common for people in Nigeria to get foodborne illnesses because of the way food is prepared in their homes while about 16% said that it was somewhat common. In contrast, 33% of the respondents reported that it was very common to get foodborne illness from restaurant food. Most of the respondents agreed on the circumstances that may lead to food-borne illnesses: not cleaning and sanitizing food equipment, utensils and food contact surfaces (92%), not washing hands before cooking or serving food (88%), eating undercooked or raw food (80%). Only 61% of the respondents believed that cutting raw meat, cooked meat and fresh fruits and vegetables on the same cutting board surface could cause food-borne illness, and only 51% of the managers indicated that allowing a sick person to cook food could lead to food-borne illness (Table 2).

**Table 2:** Circumstances that may lead to food-borne illness (% Responses).

Food Risk	Yes	No	Don’t Know
1. Eating undercooked or raw meat	80	11	9
2. Not washing hands before handling food	88	8	4
3. Cutting meat, fish, and vegetables on the same cutting board surface	61	39	0
4. The way food is cooked at home	88	8	4
5. Eating restaurant food	63	31	6
6. Not cleaning and sanitizing Equipment and utensils	92	8	0
7. Allowing a sick person to cook food	51	47	2

### 3.2. Food Handling Practices

**Figure 1:**
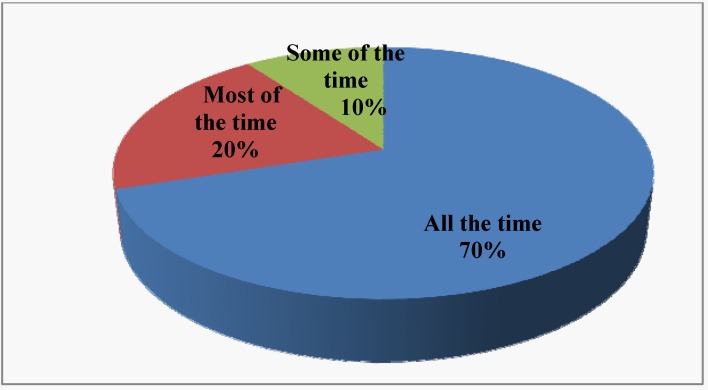
Frequency of handwashing before preparing food.

**Figure 2:**
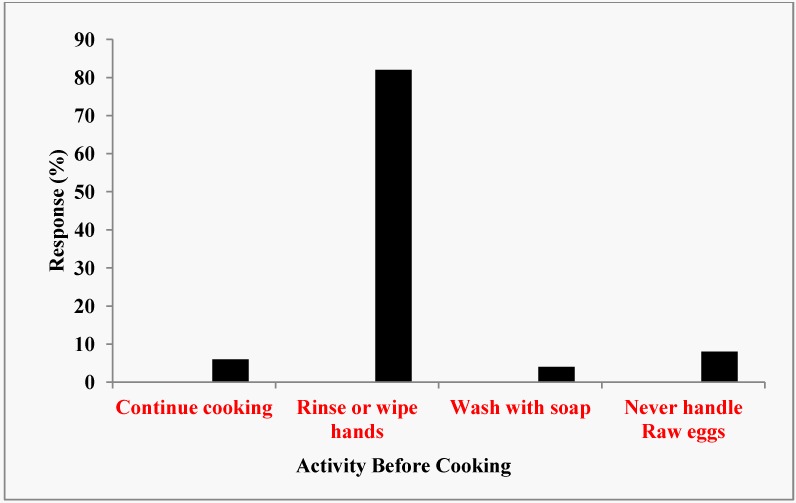
Sanitation activity after cracking raw eggs.

Seventy percent of survey respondents reported that they always washed hands with soap before preparing foods, 20% most of the time and 10% some of the time (Figure 1). About 6% of the respondents (all in class D) would continue cooking without washing, rinsing or wiping their hands after they have cracked raw eggs (Figure 2). Eighty-two percent reported that they usually rinsed or wiped their hands after cracking raw eggs. However, only 4% of the respondents (school cafeteria only) reported that they washed their hands with soap and water after cracking raw eggs before they resumed cooking. None of the respondents reported that they continued cooking after handling raw meat, raw chicken, or raw fish without first cleaning their hands. Forty-five percent (classes C and D only) reported that they rinsed or wiped their hands clean while 55% said that they washed their hands with soap before they resumed cooking. Managers that reported washing hands with soap and water before they resumed cooking by class of restaurants were 100%, 100%, 20%, and 17% for classes A, B, C, and D, respectively).

Regarding all the fruits and vegetables purchased for their restaurants, 20% of the respondents believed that all of them had been washed prior to purchase, 50% thought that most of them had been washed while 30% thought that only some of them had been washed prior to purchase. There was a wide array of methods used by the respondents to wash or rinse fruits and vegetables before using them to cook, use them for salad or serve them to customers. The methods reported included rubbing them under running water (32%), holding them under running water (3%) and, soaking them in water containing vinegar or salt (20%). After using a cutting board or other surfaces to cut raw meat, raw chicken, or raw fish, 35% reported that they rinsed the board with warm water before using the same board to cut tomato, or vegetables for salad. However, 65% reported that they washed and sanitized the board before using the same board to cut vegetables.

### 3.3. Temperature Control

**Figure 3:**
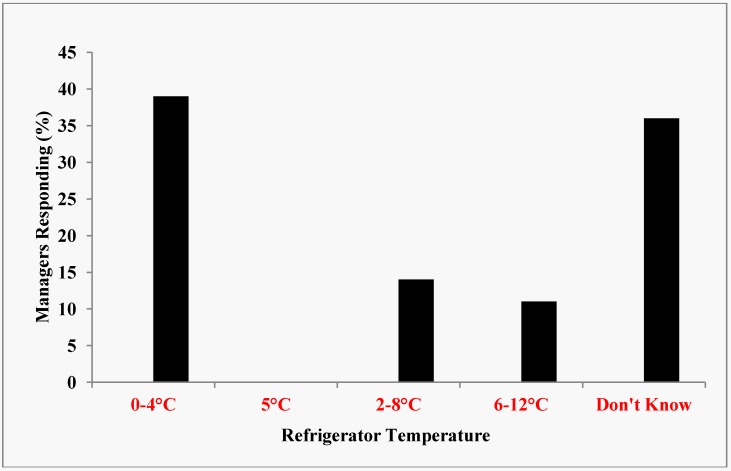
Knowledge of Ideal Refrigerator Temperature.

Because all potentially hazardous foods must be kept at an internal temperature below 45 °F (7.2 °C) or an internal temperature above 140 °F (60 °C) during display and service, it was necessary to determine if the managers of these eating places possessed the knowledge of temperature control. The United States Food and Drug Administration stipulated that the maximum temperature inside a refrigerator could be set at 41 °F or 5 °C. Most of the restaurants (90%) had at least one refrigerator, a stove and/or microwave oven at their premises. Those that did not have a refrigerator were all in Class D. Refrigerators were used to store perishable foods on a short-term basis. The main purpose of refrigeration was to slow down the growth of bacteria. The results revealed that about 38% of the restaurant managers had accurate knowledge of ideal refrigerator temperature while 36% did not know anything about refrigeration temperature (Figure 3).

After cooking food with chicken, meat or fish that would need to be saved for the next day, 15% of the respondents reported that they put it in the refrigerator immediately after cooking while 65% left the food to cool at room temperature before storing them in the refrigerator. Of the later, 55% left the food to cool at room temperature for less than two hours while 35% left it to cool for two hours or more before storing it in the refrigerator. Leftover foods were handled in different ways by the restaurants. Sixty-four percent of the respondents reported that leftover food was cooled and stored in the refrigerator, 8% allowed the food to cool and froze it while 28% discarded any food that was cooked and not eaten. None of the restaurants served refrigerated foods cold. Thirty-nine percent warmed the food below 63 °C, 22% heated the food above 63 °C while 39% did not know the proper way to reheat the food before serving.

### 3.4. Knowledge of Foodborne Pathogens

**Figure 4:**
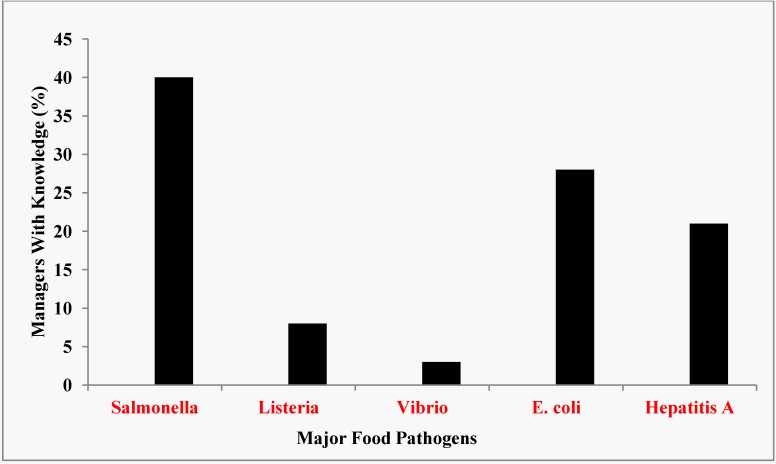
Managers’ knowledge of major food pathogens.

About 40%, 28% and 21% of the managers indicated that they had any knowledge of *Salmonella*, *E. coli,* and Hepatitis A, respectively as agents of food-borne illnesses (Figure 4). Only 8% and 3% had any knowledge of *Listeria* and *Vibrio*, respectively, as food-borne pathogens. If food contained *Salmonella* or any of the listed pathogens, 45% of the respondents reported that the food could be made safe by cooking while 22% said that the food could be made safe by adding vinegar or lemon juice to the food. Of the managers surveyed, 25% do not know how a contaminated food could be made safe while 8% reported that the food could not be made safe for eating.

### 3.5. Personal Hygiene and Sanitary Facilities/Equipment

Respondents almost unanimously (97%) agreed that their catering staff always paid close attention to personal hygiene. Only 72% of the restaurants surveyed had working toilet facilities on site. There were no toilet facilities for the convenience of both the employees and customers in 28% of the restaurants. Of the 28% restaurants without toilets, the majority were in class D (90%) while 10% were in class C. Most of the restaurants (92%) had hand washing facilities. Those that did not were all in class D.

### 3.6. Water and Materials Supply

Fifty-nine percent of the restaurants got their water from private bore holes while 41% depended on the city water supply for their water needs. The quality of water from the private bore holes had not been determined. It was also not known if this water received any treatments prior to use. Some of the restaurants also used water from the wells to prepare their foods.

The majority of the restaurants (59%) purchased their foods, ingredients and supplies daily from the open markets, 22% used regular suppliers, 14% used major registered contractors, while 5% (mostly school cafeterias) used licensed food distributors as suppliers. Flies (67%) and cockroaches (53%) were the top pests that the restaurants always encountered in their premises.

## 4. Discussion

This study evaluated the knowledge base for prevention of food-borne illnesses at foodservice operations in Owerri because disease prevention was a crucial function of food service personnel. Our foodservice survey results indicated a general lack of knowledge of the pathogens and food safety practices that were usually associated with foodborne disease outbreaks especially in tropical environment (Figure 4). In particular, these deficiencies appeared to be greatest for restaurants most likely to be used by local residents, fast food restaurants and bukaterias.

The results from the Owerri restaurant managers’ survey provide a snapshot of food safety knowledge and practice in Nigeria. From all perspectives, the results indicated that there was great need for education of foodservice personnel in Nigeria on how to prevent foodborne illnesses. The survey found that most respondents knew the circumstances that may lead to foodborne illness and the importance of hand washing. About two-thirds of the respondents understood the dangers of cross-contamination but almost half of the managers indicated that allowing a sick person to prepare or serve food could not cause people to get foodborne illness. That appeared to be one of the danger zones.

The survey found that hand washing was not practiced by the restaurant employees all the time. Only about 70% actually washed their hands all the time (Figure 1). The negative consequences of not washing hands with soap all the time prior to cooking or serving foods to customers could not be underestimated. Although 82% of the respondents indicate that they normally rinsed or washed their hands after cracking open raw eggs, only 40% and 60% of class C and D restaurants, respectively washed hands after handling open raw eggs before they resumed cooking or served foods to customers (Figure 2). Interestingly enough, none of the restaurant employees continued to cook after handling raw meat, raw chicken or raw fish. All the restaurants in classes A and B washed their hands with soap after touching raw meat, chicken and fish. Again, in classes C and D restaurants, only 20 and 17 percent, respectively, adhered to the correct hand washing protocols. One skill necessary to avoid cross contamination was to avoid using the same cutting board surface to cut raw meat, chicken or fish and fruits and vegetables. Sixty-five percent of the restaurants washed and sanitized the cutting boards each time they used them to cut raw meat, chicken or fish while 35% simply rinsed the cutting boards with warm water and used them to cut fruits and vegetables. Because cutting boards, especially those made of wood, can very easily become a source of contamination, rinsing alone even with hot water, was not sufficient to remove all the pathogens that hid in many of the tiny holes and creases of the board. This made it imperative to wash and sanitize all cutting boards after each use. This was a rule that managers must enforce in all the restaurants. Although 97% of the managers stated that their catering staff always paid close attention to personal hygiene, and 92% had hand washing facilities, this rule might be difficult to enforce in class D restaurants where 90% reported that they had no toilet facilities onsite, perhaps due to their non-permanent locations. These eating huts locally called “bukas” were usually staffed by two or three people who lack the education and training necessary to ensure food safety. Most of this type of restaurants could be termed “itinerant” because they usually move from one location to another where there are hungry people who could not afford to eat in standard and expensive restaurants. Some have permanents huts located in such places as motor parks, taxi stations, bus stations, construction sites and fields where sports activities such as soccer matches took place. Most were on wheeled carriages that could be pushed by hand. Recently, some bukas operated from a van or truck. Foods were usually cooked at home but are kept warm in insulated containers. They also have portable gas cookers for onsite cooking and warming of food. It might be difficult for this type of foodservice facilities to follow sanitation rules. In this regard, sanitation efforts could be greatly facilitated when the owners/managers were compelled to attend a crash course in sanitation and food safety as a pre-requisite for operation of their business. Also, frequent inspections (unannounced or scheduled) by government officials would make the owners put greater efforts on sanitation.

### 4.1. Education and Food Safety Knowledge

**Figure 5:**
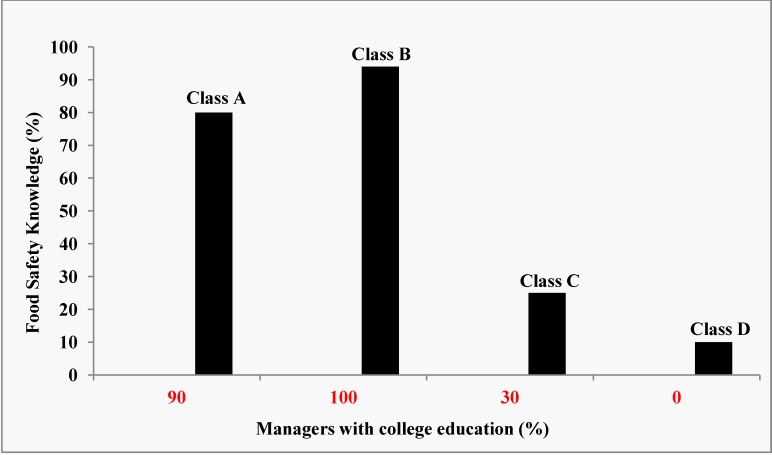
Relationship between managers’s education, restaurant class and food safety knowledge.

Based on the results of this survey, the proximate reason for the great variations in contemporary food safety knowledge and application of sanitary and hygienic standards among the restaurant managers in Owerri appeared to be the effect of training and education (Figure 5). Pearson’s Correlation Coefficient (*r* = 0.999) indicated that education was directly related to knowledge of the pathogens that caused food-borne illnesses. Training obtained by attending a Cooking School also directly affected the contemporary food safety knowledge of the restaurant managers. Managers who attended cooking school appeared to have greater knowledge of food safety than those who did not. Pearson’s Correlation Coefficient (*r* = 0.9927) indicates a linear relationship between Cooking School training and food safety knowledge. The survey also showed that managers with cooking school credentials had better food safety knowledge than those who did not attend cooking school. A plot of the responses of managers in different classes of restaurants and food safety knowledge is shown in Figure 5. Again, Pearson’s Correlation Coefficient (*r* = 8,787) indicates a very strong linear negative relationship between class of restaurants and food safety knowledge.

The results of this survey also supported the study that showed that food stall personnel had four times significantly higher odds of having poor knowledge of food safety [16]. The main reason of this was that food handlers operating food stalls or food hawking activities had low level of education and were not trained in cooking schools. Again, this assessment revealed deficiencies in attitudes, knowledge and practices in the areas of cooling/reheating, temperature control and cross contamination among the restaurant managers. It was determined that although many people did not know the basic rules of food hygiene, survey respondents had knowledge of which foods were at high risk from food poisoning, but lacked the knowledge about how a food could be made safe to eat [17].

### 4.2. Food Safety Implications

The results of this investigation presented several issues related to food safety in restaurants in Owerri. It might be difficult to ignore the fact that a significant number of foodservice workers in Owerri restaurants lacked both the education and training necessary to apply measures that would ensure the safety of foods served to their customers. In the last decades, the epidemiology of foodborne diseases changed with new or unexpected pathogens often emerging on a countrywide or worldwide scale. New foods expanded the range of potential vehicles of pathogens, wider social contexts became involved and new classes of individual became at risk [13]. This study provided some insights as to the areas of food safety that might need intensive focus during any possible training or workshops designed to educate these restaurant workers. Experts had noted that providing tailored scientifically sound and updated knowledge and identifying factors that could contribute to the generation of positive attitudes and motivate behavior change in a definite setting could help to minimize foodborne hazard and enhance the practical utility of hygiene and training for the personnel involved in foodservice functions. Several food safety issues of concern were noteworthy: hand washing, cross-contamination, temperature control, basic knowledge of pathogens of importance in food safety, personal hygiene and general food handling practices. Food safety education was an important part of extension programming because it targeted the education of consumers and food service personnel as a strategy for preventing food-borne illnesses.

### 4.3. Food and Ingredients Supply

One other area of food safety concern is how these food vendors, canteens and restaurants purchased foods, and ingredients. The survey results highlighted potential dangers of the supply sources of food and ingredients. Most of the restaurants reported that they purchased their foods and ingredients in open markets. Only school cafeterias and restaurants in major hotels had licensed suppliers. Some restaurants indicated that they depended on contractors to supply their foods and ingredients. These contractors went to the open markets on a daily basis to purchase the foods which were usually fresh, and then supplied them to the restaurants. The sanitary conditions of the foods could not be guaranteed and the open markets in Nigeria were exactly what they were called. They were located in open spaces with little or no plumbing or electricity. Butchers displayed their meats without covering them on tables while meat sellers and customers constantly touched the meat with bare hands and flies were a common sight. The hot and humid tropical environment of Nigeria was ideal for bacterial and fungal growth. It was also reported that foods and ingredients were often displayed openly on tables and on the ground in very poor sanitary environments [7]. The prevalence of flies at the markets and the apparent lack of facilities for food protection suggested a high potential for contamination. These authors also reported that unwashed fingers were normally used to feel foodstuffs and ingredients for texture and to ascertain the adequacy of manual grinding. This practice also had the potential to contribute to the microbial load of the food.

### 4.4. Education and Training

The survey results indicated that most of the restaurant managers, especially in classes C and D, did not receive the appropriate education and training on food safety and one of the factors impacting food workers’ and managers’ safe food preparation practices were food safety education and training. It had been established that workers in restaurants that provide food safety training were more likely to wash their hands when they should than workers in restaurants that did not provide this training [18]; restaurants with food safety–certified kitchen managers were less likely to be associated with foodborne illness outbreaks than restaurants without these managers and that restaurants with food safety–certified kitchen managers are less likely to have critical violations on their food safety inspections than restaurants without these managers [19]. One other convenient approach to the training of employees in food safety protocols is the “Train the Trainer” model. In this model, the manager, head chef or an educated employee undergoes the training program and is given training materials. Upon return, he/she conducts in-house training for other employees. Post-training assessments demonstrated that attendees (restaurant supervisors) increased their understanding and commitment to health and safety, and felt prepared to provide health and safety training to their employees. A study of food safety practices in assisted-living facilities in Iowa concluded that training programs, both basic and food safety and the HACCP supported improvement of safe food-handling practices and implementation of prerequisite and the HACCP programs [20]. In addition, it is important that head chefs/catering managers and other personnel in key positions to deliver essential standards in consumer food safety be supported through additional training and routine inspection to ensure that appropriate knowledge is acquired and effectively applied [21]. However, numerous studies document that education alone may not result in behavioral changes, and that to change most complex behaviors, multifaceted approaches were needed. Food safety education is most effective when messages are targeted toward changing behaviors most likely to result in foodborne illness. Some Nigerian food experts have called for continuous sensitization and training of food handlers on how to operate in hygienic environment. Their approach was to sensitize people about the enormity of the problem and then train them on how to handle food properly and safely [22]. Effective restaurant managers must supervise their staff to comply with safety and sanitary protocols involving the major control factors for pathogens. These include personal hygiene and preventing sick people from handling foods, adequate cooking (time and temperature), avoiding cross-contamination including having separate utensils to prepare foods for customers with food allergies, keeping food at safe temperatures and in clean containers, and avoiding foods from unsafe sources such as open markets. Foods, water and supplies must be obtained from sources with proven safety records. Greater manager accountability for employee noncompliance and an increased emphasis on employee education could help restaurants minimize threats to public health and strengthen the restaurant industry [23].

## 5. Conclusions

Results of this survey highlighted key prevention and safety issues that increased the likelihood of outbreaks of food-borne diseases originating from many of these restaurants. To help mitigate these risks, restaurants should be targeted for intensive inspection and improvement of the sanitary conditions of their facilities. Furthermore, the survey highlighted the potential risks of the sources of food and water supply. The lack of current knowledge of food safety among the restaurant staff in Owerri called for the development of an appropriate training program for food service personnel in order to avert the potential danger of food-borne illness outbreaks. Government involvement might include public service announcements promoting food safety and offers of some types of incentives to encourage restaurants to participate in trainings, especially fast-food restaurants and itinerant food hawking operators.

## References

[1] WHO/UNICEF Joint Monitoring Programme (JMP) for Water and Sanitation. http://www.unicef.org/wash/index_documents.html.

[2] Omotayo R.K., Denloye S.A. (2002). The Nigerian Experience on Food Safety Regulations.

[3] Oyemade A., Omokhodion F.O., Olawuyi J.F., Sridhar M.K.C., Olaesha I.O. (1998). Environmental and personal hygiene practices: Risk factors for diarrhea among children of Nigerian market women. J. Diarrheal Dis. Res..

[4] Sneed J., Strohbehn C., Gilmore S.A. (2004). Food Safety Practices and readiness to implement HACCP Programs in assisted-living facilities in Iowa. J. Am. Diet Assoc..

[5] Rheinländer T., Olsen M., Bakang J.A., Takyi H., Konradsen F., Samuelsen H. (2008). Keeping up appearances: Perceptions of street food safety in urban Kumasi, Ghana. J. Urban Health.

[6] Raab C.A., Woodburn M.J. (1997). Changing risk perceptions and food handling practices of Oregon household food preparers. J. Consum. Stud. Home Econ..

[7] Ehiri J.E., Azubuike M.C., Ubaonu C.N., Anyanwu E.C., Ibe K.M., Ogbonna M.O. (2001). Critical Control Points of complementary food preparation and handling in eastern Nigeria. Bull. World Health Organ..

[8] Osagbemi G., Abdullahi A., Aderibigbe S. (2010). Knowledge, attitude and practice concerning food poisoning among residents of Okene Metropolis, Nigeria. Res. J. Soc. Sci..

[9] Adedoyin O.T., Ojuawo A., Adesiyun O.O., Mark F., Anigilaje E.A. (2008). Poisoning due to yam flour consumption in five families in Ilorin, central Nigeria. West Afr. Med. J..

[10] Adeleke S.I. (2009). Food poisoning due to yam flour consumption in Kano (Northwest). Nigeria Online J. Health Allied Scs..

[11] Fatiregun A.A., Oyebade O.A., Oladokun L. (2010). Investigation of an outbreak of food poisoning in a resource-limited setting. Trop. J. Health Sci..

[12] World Health Organization (WHO) (2009). Global Burden of Disease.

[13] Bamaiyi P.H. (2011). Emerging food pathogens: A case study of *E. coli* 0104:H4. Cont. J. Anim. Vet.Res..

[14] Green L.B. (2013). EHS-Net restaurant food safety studies: What have we learned?. J. Environ. Health.

[15] Lando A., Verrill L. 2006 FDA/FSIS Food Safety Survey Topline Frequency Report. http://www.fda.gov/Food/FoodScienceResearch/ConsumerBehaviorResearch/ucm080374.htm.

[16] Zain M.M., Naing N.N. (2002). Socio demographic characteristics of food handlers and their knowledge, attitude and practice towards food sanitation: A preliminary report. Southeast Asian J. Trop. Med. Public Health.

[17] Raab C.A., Woodburn M.J. (1997). Changing risk perceptions and food handling practices of Oregon household food preparers. J. Consum. Stud. Home Econ..

[18] Cates S.C., Muth M.K., Karns S.A., Penne M.A., Stone C.N., Harrison J.E., Radke V. (2009). Certified kitchen managers: Do they improve restaurant inspection outcomes?. J. Food Prot..

[19] Bush D., Paleo L., Baker R., Dewey R., Toktogonova N., Cornelio D. (2009). Restaurant supervisor safety training: Evaluating a small business training intervention. Public Health Rep..

[20] Sneed J., Strohbehn C., Gilmore S.A. (2004). Food Safety Practices and readiness to implement HACCP Programs in assisted-living facilities in Iowa. J. Am. Diet Assoc..

[21] Hedberg C.W., Smith S.J., Kirkland E., Radke V., Jones T.F., Selman C.A. (2006). the EHS-Net Working Group. Systematic environmental evaluations to identify food safety differences between outbreak and nonoutbreak restaurants. J. Food Prot..

[22] AMAC (2012). Sensitization program for food vendors operating in the Abuja Municipal Area Council (AMAC). Premium Times.

[23] Dundes L., Swann T. (2008). Food safety in fast-food restaurants. J. Hum. Resour. Hosp. Tour..

